# Differential Assemblage of Functional Units in Paddy Soil Microbiomes

**DOI:** 10.1371/journal.pone.0122221

**Published:** 2015-04-21

**Authors:** Yongkyu Kim, Werner Liesack

**Affiliations:** Max Planck Institute for Terrestrial Microbiology, Marburg, Germany; American University in Cairo, EGYPT

## Abstract

Flooded rice fields are not only a global food source but also a major biogenic source of atmospheric methane. Using metatranscriptomics, we comparatively explored structural and functional succession of paddy soil microbiomes in the oxic surface layer and anoxic bulk soil. *Cyanobacteria*, *Fungi*, *Xanthomonadales*, *Myxococcales*, and *Methylococcales* were the most abundant and metabolically active groups in the oxic zone, while *Clostridia*, *Actinobacteria*, *Geobacter*, *Anaeromyxobacter*, *Anaerolineae*, and methanogenic archaea dominated the anoxic zone. The protein synthesis potential of these groups was about 75% and 50% of the entire community capacity, respectively. Their structure-function relationships in microbiome succession were revealed by classifying the protein-coding transcripts into core, non-core, and taxon-specific transcripts based on homologous gene distribution. The differential expression of core transcripts between the two microbiomes indicated that structural succession is primarily governed by the cellular ability to adapt to the given oxygen condition, involving oxidative stress, nitrogen/phosphorus metabolism, and fermentation. By contrast, the non-core transcripts were expressed from genes involved in the metabolism of various carbon sources. Among those, taxon-specific transcripts revealed highly specialized roles of the dominant groups in community-wide functioning. For instance, taxon-specific transcripts involved in photosynthesis and methane oxidation were a characteristic of the oxic zone, while those related to methane production and aromatic compound degradation were specific to the anoxic zone. Degradation of organic matters, antibiotics resistance, and secondary metabolite production were detected to be expressed in both the oxic and anoxic zones, but by different taxonomic groups. Cross-feeding of methanol between members of the *Methylococcales* and *Xanthomonadales* was suggested by the observation that in the oxic zone, they both exclusively expressed homologous genes encoding methanol dehydrogenase. Our metatranscriptomic analysis suggests that paddy soil microbiomes act as complex, functionally coordinated assemblages whose taxonomic composition is governed by the prevailing habitat factors and their hierarchical importance for community succession.

## Introduction

Comparative taxonomic profiling of microbial communities, or microbiomes, has been carried out to measure structural responses to changing environmental conditions. Using the 16S rRNA gene, correlation between microbiome composition and selective pressures were detected in various habitats with different environmental or geographic characteristics [1], [2], [3]. Microbiomes in nature exhibited interlineage associations, ranging from specific grouping of a few lineages to large assemblages with shared habitat preferences [4]. These studies showed that microbiomes possess compositional patterns that clearly deviate from a random distribution [5]. However, both theoretical and experimental community ecology and functional equivalence hypothesis between multiple species predict that identical environmental conditions can select for taxonomically different communities [6], [7]. Conservation of functional profiles among different microbiota corroborated that functional rather than taxonomic diversity represents ecological community [8]. Because both microbiome structure and function are strongly influenced by the environment, it is critical to identify the fundamental ecological processes or key factors that govern microbiome succession.

A considerable amount of bacterial genomes is dedicated to shaping the organisms’ habitats and maintaining their ecosystem [9]. Cultivation-independent genomic approaches, such as metagenomics, have greatly advanced our understanding of taxonomic and genetic diversity and ecological potential of microbial communities [10]. However, these approaches provide limited information on the functional significance of the observed genes. Metatranscriptomics, which investigates the transcriptional expression of the metagenome by large-scale RNA sequencing, gains insight into the *in situ* activities of microbiome functioning in response to environmental cues [11], [12], [13], [14]. For example, community-wide responses of marine microbial assemblages to substrate amendments (e.g., dimethylsulfoniopropionate, polyamines putrescine and spermidine) were investigated by metatranscriptomics to characterize the functional activity of members capable of utilizing these substrates [15], [16], [17]. A recent soil metatranscriptome study revealed that the degradation of the pollutant phenanthrene is initiated in accordance with the increased expression of dioxygenase, stress response and detoxification genes [18]. Furthermore, metatranscriptomics showed to be quantitative as deduced from the observation that the transformation of the herbicide atrazine proportionally increased with the abundance of transcripts implicated in the corresponding biochemical reactions [19]. Apparently, metatranscriptomics makes it possible to relate community dynamics to specific environmental cues, while gene-based approaches reveal structural and functional diversity accumulated by historical changes in environmental conditions [20].

Here, we employed metatranscriptomics to reveal structure-function relationships in microbiome succession in flooded paddy soil. Microorganisms in this ecosystem experience periodic cycles of flooding and drainage during plant cultivation. Flooding creates oxygen-limited conditions with different physico-chemical properties such as oxic surface layer, anoxic bulk soil, and rhizosphere. Distinct activities and spatial distribution of functional groups of microorganisms are established between these compartments [21], [22]. Microbial activities determine the balance between methane oxidation in the oxic surface layer and methane production in the anoxic bulk soil, resulting in net emission of this greenhouse gas to the atmosphere. Hence, paddy soil microbiomes exposed to the contrasting oxygen conditions (oxic surface layer vs. anoxic bulk soil) appeared to be a suitable model system to comparatively explore structural and functional succession driven by different environmental cues.

The relative contribution of taxonomic groups to the ribosomal metatranscriptome can be considered a function of population size and metabolic activity [23], [24]. Accordingly, Blazewicz *et al*. [25] proposed that cellularly expressed ribosomal RNA is an excellent indicator for the protein synthesis potential of microbial populations. Based on this concept, we identified the taxonomic groups that exhibited greatest protein synthesis potential during the different rice plant growth stages in flooded paddy soil. In the ripening stage, the metabolic activities in oxic and anoxic paddy soil were investigated by classifying the functional metatranscriptomes into core, non-core, and taxon-specific transcripts based on homologous gene distribution between these dominant taxonomic groups. Ubiquitous gene sets among community members differentiate the characteristics of a particular community from those of other communities, while they cannot be indicative of specific features of individual community members. Our approach allowed us to identify the ecological roles of individual taxonomic groups and biochemical coordination between community members, thereby revealing the ecological processes that drive differential microbiome succession in flooded paddy soil.

## Materials and Methods

### Rice microcosms

Paddy soil samples were provided by the Italian Rice Research Institute in Vercelli, Italy (GPS coordinates: N45.3202, E8.4186). No specific permission was required for the sampling location. Soil was taken from a drained paddy field in spring 2011 and was air dried and stored at room temperature. The soil was sieved (<2 mm) prior to use. The study did not involve endangered or protected species. Using a nylon bag to separate roots from bulk soil, rice plants (*Oryza sativa* var. KORAL type japonica) were grown in microcosms in the greenhouse as previously described by Shrestha *et al*. [26]. After transplantation of rice seedlings into the flooded microcosms, samples were taken from the surface layer (oxic zone) and the bulk soil (anoxic zone) at different incubation periods (25, 45, and 90 days). These incubation periods respectively correspond to the following plant growth stages: tillering, flowering, and ripening. The upper 2-mm surface layer was sampled using a spatula, after removal of the floodwater. Previous studies have shown that the upper 2-mm surface layer represents the oxic zone and oxygen is depleted from 200 μM at the surface layer to undetectable amounts beneath a depth of approximately 2 mm [27]. Dark gray bulk soil beneath the bottom of the nylon bag was collected, in a depth of approximately 6–8 cm. Aliquots of one gram of wet soil were transferred into 2-ml tubes and immediately shock-frozen in liquid nitrogen. Soil aliquots were stored at -80°C overnight and subjected to RNA extraction at the following day. Two independent microcosms were sampled for the 45- and 90-day incubation periods, while a single microcosm was used for the 25-day incubation period.

### Metatranscriptome library preparation

Total RNA was extracted from three soil aliquots of each of the 25- and 45-day-old microcosms using the method described by Mettel *et al*. [28]. The three aliquots were pooled, resulting in microcosm-specific extracts of total RNA for both the oxic and anoxic zones. A total of 12 soil aliquots were collected from each of the two 90-day-old microcosms and used for the extraction of total RNA, in order to have sufficient amount of RNA for mRNA enrichment. mRNA was enriched using the Ribo-Zero rRNA removal kit (Meta-Bacteria) according to the manufacturer’s instructions (Epicentre Biotechnologies, Madison, WI). The concentration of RNA was measured using Qubit RNA assay kit (Invitrogen, Eugene, OR). The integrity of total RNA and depletion of rRNA in enriched mRNA was checked by Experion RNA HighSens chips (Bio-Rad, Hercules, CA). The cDNA libraries of both total RNA and enriched mRNA were constructed by random priming using NEBNext mRNA Library Prep Reagent Set for 454 (New England BioLabs, Ipswich, MA) and tagged using the GS FLX Titanium Rapid Library MID adaptors (454 Life Sciences, Brandford, CT) according to the manufacturers`manuals. The cDNA libraries constructed from total RNA of the three different incubation periods were mixed in equal molar ratio and sequenced on the 454 GS Junior system, following the protocol of the manufacturer (454 Life Sciences). The cDNA libraries of enriched mRNA were sequenced using the 454 GS FLX instrument (454 Life Sciences) at the Max Planck Genome Centre Cologne (Germany). The raw 454 reads of each cDNA library have been deposited under the study number PRJNA215834 in the NCBI Sequence Read Archive.

### Bioinformatic analysis

The raw 454-pyrosequencing reads were preprocessed based on quality scores using PRINSEQ [29]. Low-quality reads were removed if one of the following criteria was met: length < 200 bp, mean quality score < 20, or more than 1% of ambiguous signals (N). Simple repeat sequences like homopolymers were eliminated when the complexity score, calculated by DUST algorithm, was above 7 [30]. Exact duplicate and reverse complimentary reads of the same length were defined as artificial replicates and discarded [31]. Terminal base calls at the 3’-end were trimmed off if the quality score was below 10.

Using BLASTN, the preprocessed reads derived from total RNA were screened against SILVA small and large subunit ribosomal RNA databases (SSU Ref and LSU Ref) [32], using an e-value cutoff of 1e-10. They were classified into SSU rRNA-tags, LSU rRNA-tags, or non-rRNA-tags. If reads had significant matches in both SSU Ref and LSU Ref, they were assigned according to where their best hit alignment had a higher bit score, using a custom-coded Python script. The SSU rRNA-tags were selected to determine the microbiome composition. They were grouped into operational taxonomic units (OTUs) by mapping them to sequences of the non-redundant (nr) SILVA SSU Ref database, which was dereplicated with 99% identity cutoff. Using BLAST, the mapping cutoff was 5% sequence divergence. SSU rRNA-tags mapped to the same reference sequence were grouped into the same OTU. Mapped reference sequences were selected to represent the respective OTUs. Richness estimates and diversity indices were computed using QIIME [33].

Preprocessed reads of enriched mRNA were differentiated into rRNA-tags and non-rRNA-tags as described above. The non-rRNA-tags were compared against the Rfam database using INFERNAL with default parameters to eliminate small RNA-derived reads [34]. The remaining reads were defined as mRNA-tags and subjected to BLASTX searches against NCBI nr protein database, using a bit score cutoff of 50. The annotated mRNA-tags were functionally classified into SEED subsystems and taxonomically classified based on top hit and the lowest common ancestor (LCA) using MEGAN4 [35]. Statistical analysis based on functional profiles was done using STAMP software package [36]. Taxon-specific protein databases were constructed by searching the NCBI nr protein database with name of a particular taxon in the organism field. The mRNA-tags were compared by BLASTX independently to each taxon-specific database using a bit score cutoff of 50. The BLAST outputs were parsed using custom-made Python scripts to classify the mRNA-tags into core, non-core, and taxon-specific data sets on the basis of the presence or absence of homologous genes in the taxon-specific databases (S1 and S2 SuppInfo). The mRNA-tags that had no homolog in any of the taxon-specific protein databases were subjected to BLASTX search against the NCBI nr protein database for taxonomic classification.

## Results and Discussion

### Basic statistics of metatranscriptome sequencing

Total RNA extracted from the oxic and anoxic zones of flooded rice microcosms was analyzed by 454-pyrosequencing at different incubation periods (25, 45 and 90 days). This approach aimed at identifying the microbial groups contributing most to the cellularly expressed rRNA pools. Across all samplings, a total of 35,851 and 27,304 reads of small subunit ribosomal RNA (SSU rRNA-tags) were obtained for the oxic and anoxic zones, respectively. The average read lengths ranged from 423 bp to 469 bp (S1 Table). Microcosms with rice plants in the ripening stage (90 days) were used to comparatively explore the *in situ* microbiomes`functioning. 454-pyrosequencing of enriched mRNA resulted in 73,974 and 464,906 raw reads from the oxic and anoxic zone, respectively. Reads derived from rRNA and small non-coding RNA were excluded from further analysis. After processing of the raw data, 48,816 reads (oxic zone) and 92,245 reads (anoxic zone) were classified as protein-coding transcripts (mRNA-tags) (S2 Table).

### The microbiomes' taxonomic compositions

The random SSU rRNA-tags were clustered into OTUs using a sequence identity cutoff of 95%. Rarefaction curves revealed that, on average, 35% of the estimated genus richness was covered (S1A Fig). About 20% of SSU rRNA-tags could not be mapped using the 95% identity cutoff and were identified to represent novel genera. However, the decrease of the sequence identity cutoff to 90% and 85% increased the mapping efficiency up to 98% (S1B Fig). This clearly showed that microbial diversity at higher taxonomic ranks, such as family or order level, is confidently explored by the given set of SSU rRNA-tags.

**Fig 1:**
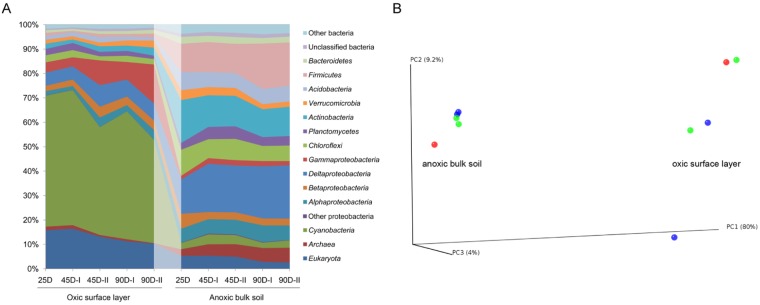
Phylum-level changes in microbiome composition in the oxic and anoxic zones over incubation period (A) and principal coordinate analysis based on the weighted UniFrac distance matrix (B).

**Fig 2:**
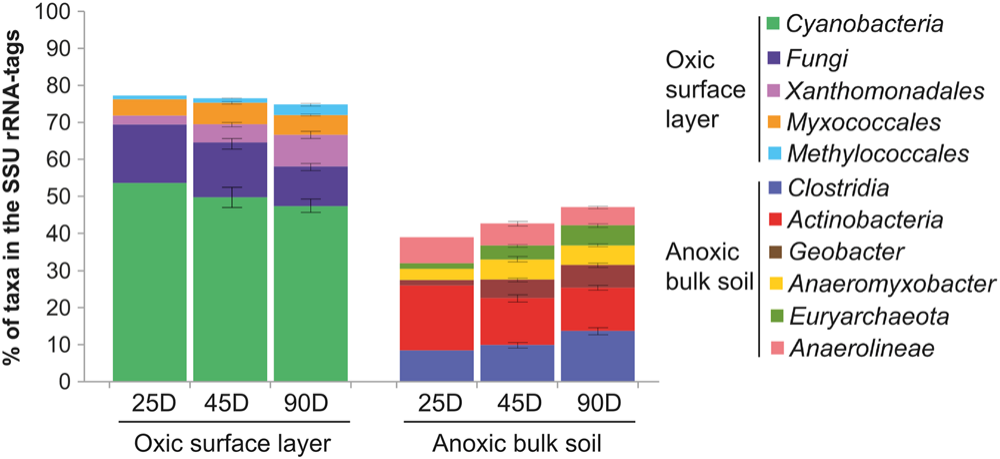
Temporal changes in the relative abundance of the dominant taxonomic groups in the SSU rRNA-tags over incubation period. Each of the dominant groups persistently contributed > 5% to total SSU rRNA-tags or showed significant fold changes in their relative abundance during incubation, while no other taxonomic group accounted for greater than 3% at order level. The abundance cutoff was applied to the lowest taxonomic rank possible, explaining why the taxonomic ranks vary from genus to phylum levels.

The taxonomic composition of the microbiomes established in the oxic and anoxic zones greatly differed, even at phylum level (Fig 1A). Spatial separation between the oxic surface layer and anoxic bulk soil explained more than 70% of the compositional variance, as evidenced by principal coordinate analysis (Fig 1B). The effect of incubation time was less than 9%, thereby suggesting that the community composition was stable over the different plant growth stages. Despite the high taxon richness, the microbiomes in both oxygen zones were dominated by a limited number of taxonomic groups. Members of the *Cyanobacteria*, *Fungi*, *Xanthomonadales*, *Myxococcales*, and *Methylococcales* were the most abundant and metabolically active taxa in the oxic zone, while *Clostridia*, *Actinobacteria*, *Geobacter*, *Anaeromyxobacter*, *Anaerolineae*, and *Euryarchaeota* were most prevalent in the anoxic zone. Each of these taxa persistently contributed > 5% to the total SSU rRNA-tags or showed significant fold increase in their relative abundance over incubation time (Fig 2). They will be referred to as the dominant groups. Collectively, they accounted for up to 75% (oxic zone) and 50% (anoxic zone) of total SSU rRNA-tags, suggesting that they occupied crucial roles in microbiome functioning related to their high protein synthesis potential [25]. Representative groups that contributed < 3% to the total SSU rRNA-tags at the order level were as follows: *Acidimicrobiales*, *Burkholderiales*, *Sphingobacteriales*, and *Verrucomicrobiales* in the oxic zone, and *Acidobacteriales*, *Bacillales*, and *Syntrophobacterales* in the anoxic zone. Some order level groups were detected in both oxygen zones, including *Rhizobiales* and *Rhodospirillales*. Highly similar taxonomic patterns between biological replicates corroborated experimental reproducibility of RNA extraction and sample-specific analysis of total RNA by 454-pyrosequencing. Due to the near-steady-state community organization of major taxa throughout incubation period, functional succession in the oxic and anoxic zones was assessed only in 90-day-old microcosms using combined libraries of two biological replicates.

### SSU rRNA versus mRNA: a taxonomic perspective

**Fig 3:**
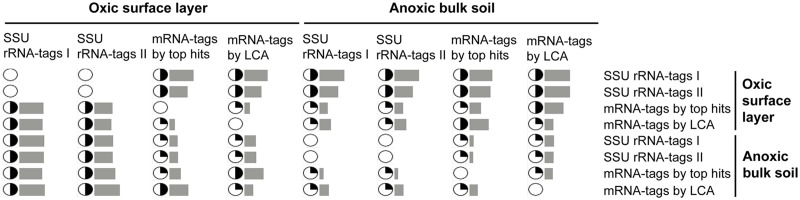
Discrepancies between the taxonomic patterns derived from SSU rRNA-tags and mRNA-tags. Bars indicate the measures of Bray-Curtis dissimilarity in pairwise comparison of taxonomic composition. Minimum value (0) means the identical composition and maximum value (1) shows there is no shared taxonomy. Filling circles indicate the range of Bray-Curtis indices by quarter.

**Fig 4:**
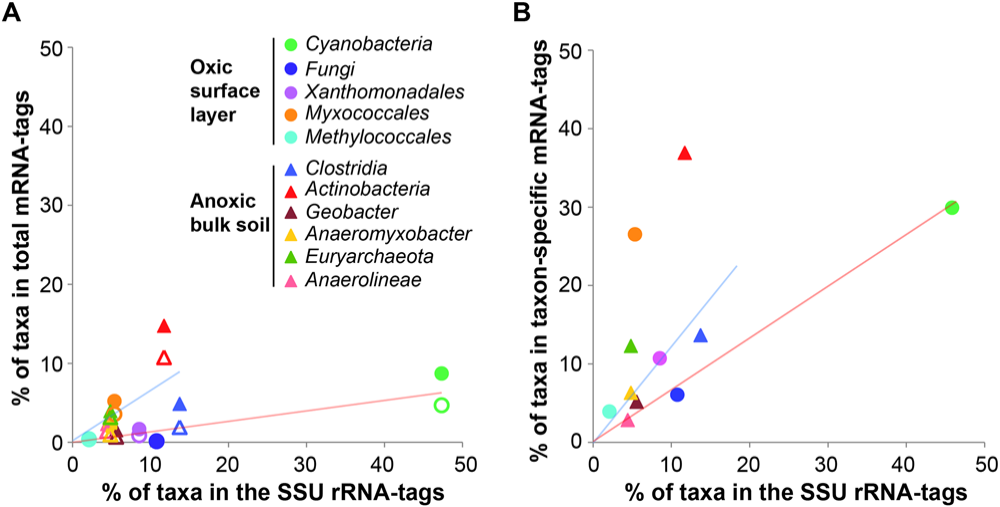
The relative contribution of the dominant taxonomic groups to the community composition (x-axis: SSU rRNA-tags) and microbiome function (y-axis: mRNA-tags). (A) The top hit (filled symbols) and LCA (unfilled symbols) of BLAST hits were taken for conventional taxonomic assignment of total mRNA-tags. (B) The relative abundance of the taxon-specific mRNA-tags for each of the dominant groups was normalized to that of the entire microbiome by taking into account the proportion of the dominant group-derived mRNA-tags on total mRNA-tags (see Fig 5). Linear regression curves were generated with intercept value of 0 for the oxic zone (red line) and anoxic zone (blue line).

In a conventional approach, the taxonomic origin of mRNA-tags was deduced from the (i) top hit and (ii) lowest common ancestor (LCA) of maximum 100 BLASTX hits in NCBI nr protein database (S2 Fig). We made an attempt to quantify the discrepancy between the taxonomic patterns derived from SSU rRNA and mRNA. Compositional dissimilarity between the taxonomic patterns was calculated at the phylum level using Bray-Curtis indices (Fig 3). Unexpectedly, the taxonomic composition of the mRNA-tags from the oxic zone was more dissimilar to its corresponding rRNA-tags than to the taxonomic compositions of the rRNA-tags and mRNA-tags from the anoxic zone. Overall, the patterns were not separated according to the oxic or anoxic zone, although mRNA-tags and SSU rRNA-tags of each zone were obtained from the same extract of total RNA. Additionally, there was only a weak correspondence between the relative abundances of the dominant groups in the rRNA and mRNA data sets (Fig 4A). Except for *Actinobacteria*, all the dominant groups were highly underrepresented in mRNA-tags relative to their contribution to rRNA-tags. In fact, these taxonomic discrepancies are inevitable due to the following reasons: (i) taxonomic bias of mRNA-tags towards phyla that are overrepresented by many sequenced genomes [37], (ii) horizontal gene transfer events between phylogenetically distant microorganisms [38], and (iii) high sequence conservation in house-keeping genes and different evolutionary rates among functional groups of proteins [39]. However, a significant underrepresentation of *Cyanobacteria* in protein-coding transcripts has also been observed in the metatranscriptomes of marine waters and photosynthetic microbial mats, despite the fact that the *Cyanobacteria* residing in these environments are well represented by genome and metagenome sequences [40], [41]. The cyanobacterial example implies that incongruent representation of taxa between rRNA and mRNA pools could have reasons other than difficulties in taxonomic classification of mRNAs. To address the question of underrepresentation, we assessed how widely homologs of the expressed genes are distributed among the dominant groups and aimed to extract mRNA-tag data sets that can be specifically assigned to particular taxonomic groups.

### Defining core, non-core and taxon-specific mRNA-tags

On the basis of their contribution to the SSU rRNA-tag data sets, the protein synthesis potential of the dominant groups was estimated about 75% and 50% of the total microbiome in the oxic and anoxic zone, respectively. Given that (i) 10^8^ to 10^9^ bacterial cells are present in one gram of paddy soil [42], (ii) bacterial cells contain a number of genes ranging from about 500 to 9000 and (iii) multiple transcript copies are being produced from most genes in active cells [43], we anticipated that the mRNA-tags obtained from a major, but still limited, sequencing effort will be derived primarily from the dominant groups. Analysis of the transcriptional activity of the low-abundant taxanomic groups was not the objective of this study and would have required a much greater sequencing effort. Therefore, instead of searching against NCBI nr protein database, we first compared mRNA-tags to the taxon-specific protein databases that had been constructed for each of the dominant groups (Table 1). Overlaying the annotation profiles of the taxon-specific protein databases classified mRNA-tags into core, non-core and taxon-specific mRNA-tags based on homologous gene distribution (S3 Fig). The mRNA-tags shared between all of the dominant groups or, at least, all bacterial members were defined as core mRNA-tags. Confinement to only bacterial members was made because sequence variations among the three domains of life may be too great to detect all gene homologs [44]. Among the non-core mRNA-tags, those unique to only one of the dominant groups were defined as taxon-specific mRNA-tags.

**Table 1:** Size of the taxon-specific protein databases derived for the dominant groups from NCBI nr protein database and the number of mRNA-tags that showed BLASTX hits to the respective database.

	Taxon	Number of proteins in taxon-specific database	Number of annotated mRNA-tags and their relative proportion of total annotated mRNA-tags
**Oxic surface layer**	*Cyanobacteria*	680,891	18,768 (55.2%)
	*Fungi*	2,525,420	10,189 (30.0%)
	*Xanthomonadales*	360,722	17,050 (50.2%)
	*Myxococcales*	185,417	19,821 (58.3%)
	*Methylococcales*	40,965	13,741 (40.4%)
**Anoxic bulk soil**	*Clostridia*	1,709,159	15,903 (52.6%)
	*Actinobacteria*	3,817,624	17,903 (59.3%)
	*Geobacter*	76,981	11,314 (37.4%)
	*Anaeromyxobacter*	36,566	10,455 (34.6%)
	*Euryarchaeota*	580,253	11,814 (39.1%)
	*Anaerolineae*	6,335	8,167 (27.0%)

Note that most of the mRNA-tags had multiple taxonomic hits. The percentage values in parentheses indicate the proportion of total annotated mRNA-tags having homologous genes in the individual taxon-specific protein databases. Their homologous gene distributions were visualized in an Edwards-Venn diagram (S3 Fig).

**Fig 5:**
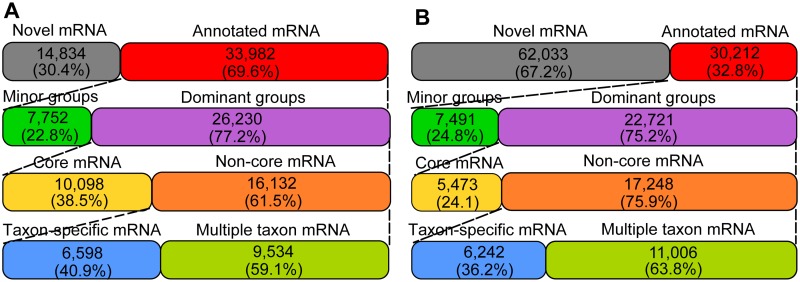
The hierarchical classification profile of putative mRNA-tags retrieved from the oxic (A) and anoxic (B) zones. Novel mRNA-tags had no homologous proteins in NCBI nr protein database. Among annotated mRNAs, those having no homologs with any of the dominant groups were indicated to be derived from minor groups. They were searched against NCBI nr protein database to infer taxonomic origin.

Among the mRNA-tags functionally annotated, 77.2% (oxic zone) and 75.2% (anoxic zone) had homologs with one or several of the dominant groups (Fig 5). The normalized representation of the dominant groups was prominently increased among the taxon-specific mRNA-tags relative to their abundance among SSU rRNA-tags (Fig 4B), suggesting that the protein synthesis potential of a particular taxonomic group within a complex community is well reflected by the expression level of genes unique to that group. The increased representation of the dominant groups in taxon-specific mRNA-tags is assumed to be due to the exclusion of highly conserved genes of uncertain taxonomic origin and of most horizontally transferred genes. Given the sequencing effort applied, our study presumably provides an insight into the transcripts highly expressed by the dominant groups in the two replicate microcosms used for functional metatranscriptomics. Analysis of single composite samples by 454-pyrosequencing has been applied in various studies to compare community-wide transcriptional activity between different environmental conditions [18], [45].

### Functional profiles of core and non-core mRNA-tags

**Fig 6:**
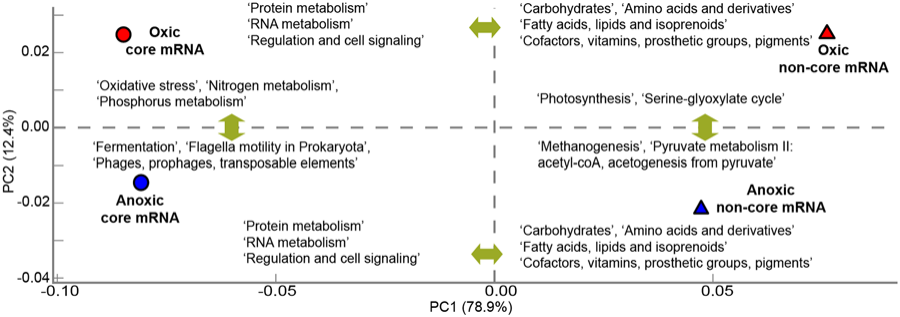
Principal component analysis of the core mRNA-tags (circle) and non-core mRNA-tags (triangle) based on the functional expression profiles using SEED subsystems. Samples indicated in red and blue relate, respectively, to the oxic and the anoxic zone. Differential expression of subsystems between two samples was calculated using two-sided Chi-square test with Yate's correction. Level 1 subsystems which were most significantly overrepresented in either core or non-core mRNA-tags (*P*-value < 0.01) are shown in a horizontal comparison. Level 2 subsystems showing differential expression between the oxic and anoxic zones are displayed by vertical comparison.

The core mRNA-tags accounted for 38.5% and 24.1% of the dominant group-derived mRNA-tags in the oxic and anoxic zone, respectively (Fig 5). Both predicted function and alignment position were highly conserved for most core mRNA-tags among their homologs, although some homologs were annotated as hypothetical proteins. Apparently, the core mRNA-tags were expressed from highly conserved ubiquitous genes, representing functional features exhibited by all the dominant groups in the respective oxygen zone. We examined the functional profiles of the core mRNA-tags using SEED subsystem-based annotation and compared them with those of the non-core mRNA-tags. The core mRNA-tags mostly encoded basic cellular machineries, such as protein and RNA metabolism and regulation and cell signaling, while the non-core mRNA-tags were more frequently implicated in carbohydrate, amino acids and derivatives, and lipid metabolisms (Fig 6). Principal component analysis, however, showed that the functional profiles of the core mRNA-tags remarkably differed between the two microbiomes. Essential capabilities to adapt to either aerobic or anaerobic conditions were considerably represented in the core mRNA-tags, but differentially expressed between the oxic and anoxic zones (e.g., oxidative stress vs. fermentation). Taken together, the core mRNA-tags were revealed to encode not only basic cellular machineries but also cellular functions that are indispensible to be metabolically active under the prevailing conditions. Differential expression of core features between the two microbiomes pointed towards different oxygen availability as a key determinant for structural succession.

**Fig 7:**
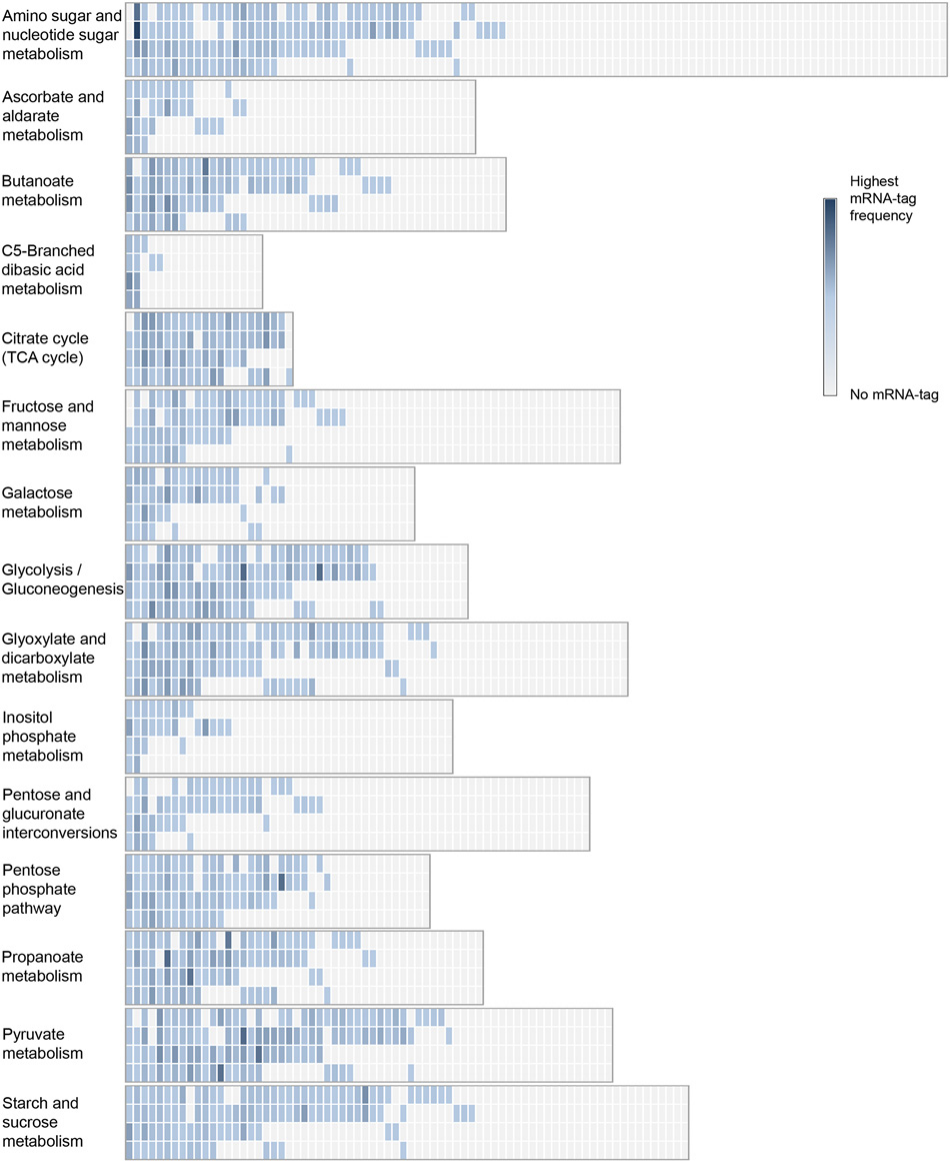
Heat map of KEEG pathways belonging to the carbohydrate metabolism represented by core and non-core mRNA-tags of the oxic and anoxic zones. Each column represents an enzyme responsible for catalytic reactions of the individual pathways. The first two rows show the expression patterns of the non-core mRNA-tags in the order oxic vs. anoxic zone. The next two rows represent the core mRNA-tags in the same order.

Carbon bioavailability is one of the primary factors affecting composition and activity of soil microbiomes [46], [47]. A large fraction of the mRNA-tags related to carbohydrate metabolism was classified into non-core mRNA-tags. In general, a wider range of enzymatic reactions that, within the KEGG pathways, belong to carbohydrate metabolism were affiliated with non-core mRNA-tags than with core mRNA-tags (Fig 7). As shown in the following sections, knowledge of the distribution of non-core mRNA-tags among the dominant groups provides insight into how soil microbiomes vary in functional complexity and maintain their heterotrophic lifestyle using a variety of carbon sources as substrates. Enzymatic reactions involved in central carbon metabolism including ‘glycolysis/gluconeogenesis’, ‘pyruvate metabolism’, and ‘citrate cycle’, however, were detected in the core-mRNA-tags with higher coverage and quantity than other pathways of carbohydrate metabolism. These core pathways are highly conserved and universally present in most organisms to generate ATP from glucose metabolism or to serve for anaplerotic reactions. In addition to carbohydrate metabolism, SEED subsystems related to carbon cycling, such as ‘photosynthesis’, ‘serine-glyoxylate cycle’, ‘methanogenesis’, and ‘pyruvate metabolism II: acetyl-coA, acetogenesis from pyruvate’, were differentially represented between the non-core mRNA-tags of the oxic and anoxic zones (Fig 6). These observations substantiate that carbon sources affect microbiome composition by activating or enriching groups of microorganisms able to utilize particular substrates.

### Taxon-specific mRNA-tags from the oxic zone

**Fig 8:**
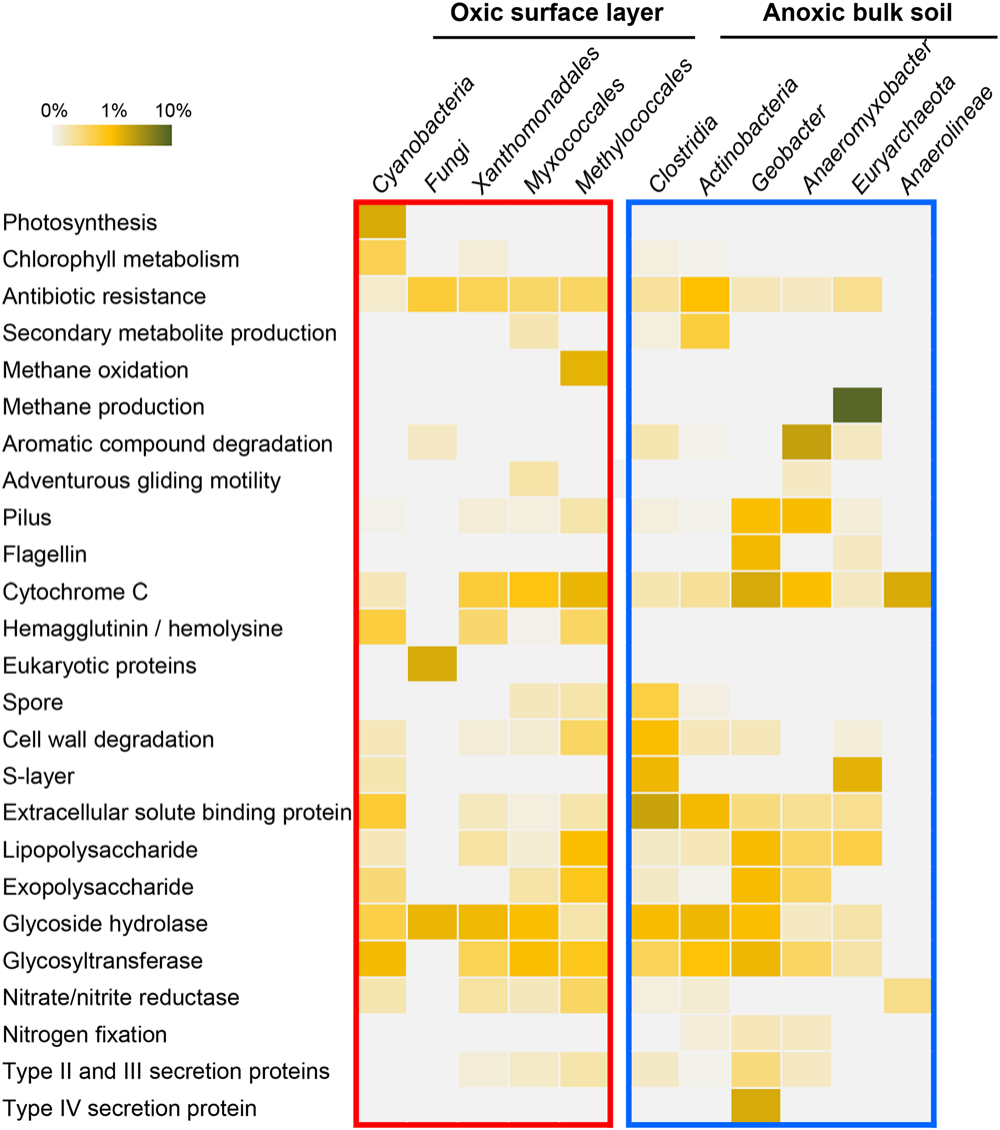
The expression of key functions in each dominant group, as derived from the analysis of the taxon-specific mRNA-tags. The relative expression level of key functions within each group was calculated by dividing the number of taxon-specific mRNA-tags involved in the respective function by the total number of taxon-specific mRNA-tags determined for the respective group.

A large fraction of the *Cyanobacteria*-specific mRNA-tags was related to chlorophyll metabolism and photosystem I and II, revealing high photosynthesis activity under light (Fig 8). Transcripts for nitrate ABC transporter and nitrate/nitrite reductases suggest that *Cyanobacteria* assimilated nitrate as an inorganic nitrogen source [48], [49]. The cyanobacterial assemblage was mainly composed of small unicellular *Synechococcus* spp. Both prevalence of *Synechococcus* and functional annotation of the taxon-specific transcripts indicate that *Cyanobacteria* coupled oxygenic photosynthesis with nitrate cycling. Photosynthetic activity agrees well with previous findings of increased O2 concentrations during the daytime, fulfilling the high biological oxygen demand for methane oxidation and organic matter degradation in the oxic zone [50]. In addition, the expression of genes encoding exopolysaccharide synthesis and proteins for polysaccharide translocation demonstrates that carbon fixed by photosynthesis was converted into polysaccharides and presumably supplied to other community members.

Members of the *Myxococcales* mainly belonged to *Polyangiaceae*, *Nannocystaceae*, and *Haliangiaceae* as derived from the SSU rRNA-tags, implying a saprophytic life style [51], [52]. Taxon-specific expression of various cellulolytic enzymes, including glucanase, amylase, glucosidase, chitosanase, and xylosidase, as well as N-acetylmuramoyl-L-alanine amidase that hydrolyzes amide bonds in cell wall peptidoglycan agrees well with their known capabilities of feeding on cells and recycling carbon and nitrogen from biomolecules under nutrient limitation in soil [53]. Secondary metabolite production, such as polyketides and insecticidal toxin complex, was found to be active only among members of the *Myxococcales*, while antibiotic resistance activity was observed for all of the dominant groups. Polyketide is a secondary metabolite of various structural types that can be transformed into antibiotics (e.g., rapamycin, erythromycin, and lovostatin), underlining the potential of the soil microbiome to be a reservoir of bioactive molecules [54].

One of the biogeochemical characteristics in the oxic zone is methane oxidation. The amount of methane that is oxidized prior to emission to the atmosphere, is in the range of 45% to 60% of the methane produced in the anoxic zone [55]. Despite the relatively minor contribution of *Methylococcales* to the SSU rRNA-tags of the dominant groups, a high transcript abundance of genes encoding particular methane monooxygenase and methanol dehydrogenase was detected. High gene expression of ecologically relevant processes performed by low-abundant organisms was frequently observed in natural environments. For example, *Crenarchaeota* were found to be very active in ammonia oxidation as derived from the normalized expression levels of *Crenarchaeota-*related *amt* and *amoABC* genes [56].

Transcripts encoding filamentous hemagglutinin and xylose isomerase were frequently detected among the *Xanthomonadales-*specific mRNA-tags. Hemagglutinin is involved in plant tissue attachment and biofilm formation [57]. Xylose isomerases catabolize xylan, which is the main building block of hemicellulose. The co-expression of these genes suggests that *Xanthomonadales* play a direct or indirect role in the decomposition of plant material, presumably in concerted action with other cellulose-degrading organisms such as *Fungi*. As deduced from forest soil eukaryotic metatranscriptome data [45], *Fungi* expressed enzymes involved in the turnover of plant biomass such as pectin depolymerase, xylosidase, and glycoside hydrolases. Signature transcripts of eukaryotic organisms, like those encoding myosin and splicing factor, were detected exclusively in our *Fungi*-specific mRNA-tags.

### Metabolic interactions revealed by non-core mRNA-tags

Members of the *Xanthomonadales* have frequently been found in relatively high abundance in environments where methane oxidation occurs such as peatlands and fruitland overlying a coal-bed methane seep [58], [59]. In our study, most of the SSU rRNA-tags affiliated with *Xanthomonadales* had the highest sequence identity with an uncultured bacterial 16S rRNA gene, which was obtained from stable isotope probing experiments using ^13^CH_4_ as a substrate [60]. The relative abundance of the *Xanthomonadales*-related SSU rRNA-tags increased with incubation period, concomitantly with those of *Methylococcales* (Fig 2). These observations led us to speculate that there may be a metabolic link between these two groups. Taxon-specific mRNA-tags did not provide an explanation for the concurrent occurrence of *Xanthomonadales* with methane-oxidizing bacteria. However, transcripts for methanol dehydrogenase shared exclusively by *Methylococcales* and *Xanthomonadales* were detected in the non-core mRNA-tags, suggesting *in situ* methanol oxidation activities of both taxa. As methanol is the first metabolic intermediate of methane oxidation by methanotrophic bacteria, this homolog distribution pattern of methanol dehydrogenase transcripts allows us to assume that members of the *Xanthomonadales* feed on methanol produced by and released from *Methylococcales*. Such cross-feeding of carbon via methanol or formate has previously been observed to occur between methanotrophs and methylotrophs [61]. Microorganisms involved in cross-feeding would require homologous genes to utilize the shared metabolic intermediates. Investigation of non-core, but not taxon-specific, mRNA-tags could provide insights into the metabolic network and/or food chain transfer in complex microbiomes.

### Taxon-specific mRNA-tags from the anoxic zone

Under anaerobic condition in the dark, different microbial guilds interact to decompose complex organic matters to methane, and microorganisms gain energy by anaerobic respiration or fermentation [62]. The taxon-specific mRNA-tags should allow us to identify particular roles of the dominant groups in the anaerobic food chain (Fig 8). The *Actinobacteria-*specific mRNA-tags encoded diverse cellulolytic enzymes, such as cellulase, xylosidase, cutinase and lignin peroxidase, as well as glycoside hydrolase. Among the mRNA-tags derived from the minor taxonomic groups (< 3% of SSU rRNA-tags), multiple transcripts for acetyl xylan esterase were affiliated with *Acidobacteria*. The esterase activity leads to the solubilization of xylan [63]. Thus, it is reasonable to assume that *Actinobacteria* and *Acidobacteria* play important roles in the decomposition of lignocellulosic materials in plant cell walls. In addition, transcripts involved in antibiotic production, including lantibiotic modifying enzyme and polyketide synthase, were expressed in the anoxic zone only by *Actinobacteria*. Notably, *Actinobacteria* in the anoxic zone and *Myxococcales* in the oxic zone occupied comparable functional roles, being on the one hand primary degraders of complex organic matters and on the other hand secondary metabolite producers (S4 Fig).

Among the *Clostridia*-specific mRNA-tags, transcripts encoding diverse types of glycoside hydrolase as well as cellulosome components, including cellulosome-anchoring protein and endoglucase, suggest cellulose degradation activity. Fermentation activity was also revealed by high abundance of transcripts for sugar ABC transporter extracellular solute binding protein. Clostridial species, like *C*. *acetobutylicum* and *C*. *thermocellum*, transport sugars into cells by either phosphoenolpyruvate(PEP)-dependent phosphotransferase system or ATP-dependent transporter system [64], [65]. Although many transcripts related to fermentation pathways were present in the core mRNA-tags, high taxon-specific expression of sugar transport systems indicates that degradation products from cellulose hydrolysis were utilized primarily by *Clostridia*, rather than by other dominant groups. High abundance of S-layer transcripts suggests ongoing cell proliferation and relates well to the gradual increase of *Clostridia* among the SSU rRNA-tags with incubation period (Fig 2). Given that the production of S-layer proteins is energetically expensive [66], *Clostridia* were metabolically highly active, corroborating that this group is of significant importance for the anaerobic breakdown of polymers in flooded paddy soil.

In anoxic paddy soil, the oxidation of acetate by *Geobacter* is coupled with the dissimilatory reduction of iron [67], [68]. Transcripts encoding type IV pili and type IV pilus secretin were homologous to unique gene sequences from *Geobacter metallireducens*, whose expression products are involved in extracellular electron transfer [69], [70]. The notable abundance of flagellin and cytochrome C transcripts indicated the motility of *Geobacter* spp. to access and reduce Fe(III) oxide [71]. The functional expression profile of the *Anaeromyxobacter-*specific mRNA-tags obviously differed from that of *Myxococcales* in the oxic zone (Fig 8, S4 Fig). While *Anaeromyxobacter* exhibited strong transcript activity in aromatic compound degradation, predation and secondary metabolite production activities that are typical of other myxobacteria were not detected. The expression of different types of motility was also evident. Type-IV pilus-based social (S-) motility and flagellum-mediated motility was highly expressed by *Anaeromyxobacter*, whereas transcripts encoding adventurous (A-) gliding motility was a characteristic of the myxobacteria inhabiting the oxic zone.

Most of the SSU rRNA-tags derived from *Euryarchaeota* were affiliated with methanogenic archaea. The most abundant transcript species among the *Euryarchaeota*-specific mRNA-tags encoded methyl coenzyme M reductase (MCR), which catalyzes the terminal step of methane formation. Most MCR transcripts were derived from *Methanosarcina* and *Methanosaeta*, the two different types of acetoclastic methanogens. Additionally, all archaeal S-layer protein transcripts were derived from *Methanosaeta* spp. (S5 Fig). Both the gradual increase in the relative abundance of SSU rRNA-tags related to *Clostridia* and acetoclastic methanogens with incubation period and the functional annotation of their taxon-specific mRNA-tags suggest a close metabolic link between the two groups, with acetate as the key metabolic intermediate [71]. Putative roles of *Anaerolineae* could not be inferred, primarily due to the high abundance of hypothetical proteins (~63%) among their taxon-specific transcripts.

## Concluding Remarks

Metatranscriptome data of natural microbial communities are largely derived from highly conserved genes which are involved in basic cellular maintenance [72]. Due to the high sequence conservation and ubiquity, transcripts of these genes are frequently affiliated with incorrect taxonomy. Their high abundance in metatranscriptomes makes it difficult to detect ecologically significant expression of genes that are present in low abundance in metagenomes. This is despite the fact that the expression level of such genes, normalized to gene abundance within community, is significantly higher than that of highly conserved genes [72]. Classification of transcripts based on homologous gene distribution allowed us to identify the functional signatures of active populations and the metabolic contributions of individual taxa to community-wide functioning. This approach provided insights into the ecological process of microbiome succession in response to the prevailing conditions.

*Cyanobacteria*, *Fungi*, *Xanthomonadales*, *Myxococcales*, and *Methylococcales* were the most abundant and metabolically active groups in the oxic zone, while *Clostridia*, *Actinobacteria*, *Geobacter*, *Anaeromyxobacter*, *Anaerolineae*, and methanogenic archaea dominated the anoxic zone. They were stably maintained throughout the incubation period. Distinct taxonomic composition between the two oxygen zones was related to the expression of different sets of highly conserved and ubiquitous genes, whose expression is essential for metabolic activity in the respective zone. The differences in the core features between the two microbiomes suggest that different oxygen availability is the necessary condition in determining structural succession.

Although metabolic traits and most substrate utilization capacities are known to be highly redundant among microbial groups, we observed limited functional redundancy among the dominant groups of either oxic or anoxic zone. Some functions were identified to be expressed in both the oxic and anoxic zones, but by different taxonomic groups. For instance, polymer degradation and secondary metabolite production were performed by either *Myxococcales* (oxic zone) or *Actinobacteria* (anoxic zone). While *Fungi* and *Xanthomonadales* were suggested to cooperate in the decomposition of plant materials, most of the dominant groups in the anoxic zone collaboratively transformed organic matter. These functions were maintained in both microbiomes, given that plant materials are one of the major carbon sources in soil and antibiotics are essential to manage interaction networks within a natural microbial community [73]. Preselection of active populations by different oxygen availability results in distinct sets of taxa that carry out similar functions during successional change in the two oxygen zones. This finding corroborates that microbiome functioning cannot be determined solely on the basis of taxonomic composition or genetic potential [74], [75].

By contrast, photosynthesis and methane oxidation carried out respectively by *Cyanobacteria* and *Methylococcales* were unique to the oxic zone. Methane production by methanogenic archaea and aromatic compound degradation by *Anaeromyxobacter* was a characteristic of the anoxic zone. Nutrient or energy sources that are exclusively or majorly available in one of the two zones, such as light in the oxic surface layer or acetate in the anoxic bulk soil, induced the activities of particular taxonomic groups which are capable of assimilating or metabolizing these resources. While oxygen led to a prescreening of adaptable taxa, nutrient and energy sources determined the expression of metabolic diversity on spatial and temporal scale. Each of the dominant groups was revealed to serve as a functional unit activated by a relevant energy or nutrient source. Microorganisms embedded in a complex community exhibit different gene expression profiles towards energetically favored or taxonomically specialized metabolic reactions, partly owing to metabolite exchange. Systematic assemblages of functional units benefit from metabolic networks to access resources and niches that cannot be occupied by the activity of individual groups.

## Supporting Information

S1 Fig OTU-based microbial richness estimation for samples from the oxic surface layer and anoxic bulk soil (A) and the mapping efficiency of the SSU rRNA-tags at different taxonomic ranks (B). Samples from the oxic and anoxic zones of the same incubation time point are indicated in the same color but differentiated by dashed and solid lines, respectively. Note that some lines are superimposed.
(TIF)

S2 Fig Comparison of the phylum-level taxonomic patterns derived from SSU rRNA-tags and mRNA-tags for the oxic (A) and anoxic (B) zones. (TIF)

S3 Fig Edwards-Venn diagram showing the distribution of mRNA-tags unique to, or shared by, the dominant microbial groups in the oxic (A) and anoxic (B) zones. (TIF)

S4 Fig Hierarchical clustering of the dominant taxonomic groups based on the expression profiles of the taxon-specific mRNA-tags (Fig 8). (TIF)

S5 Fig Taxonomic composition of methanogenic archaea based on SSU rRNA-tags (A-C) and mRNA-tags encoding methyl coenzyme M reductase (*mcr*) (D) and S-layer protein(s) (E). (TIF)

S1 SuppInfo A Python script parsing BLAST xml outputs to extract alignment profiles. (PY)

S2 SuppInfo A Python script to classify putative mRNA-tags into core, non-core, and taxon-specific transcripts based on homologous gene distribution. (PY)

S1 Table Statistics of ribosomal metatranscriptome libraries. Total RNA was extracted from two independent replicate microcosms, except for the 25-day incubation period. The proportion of exact duplicates and preprocessed reads is given in parenthesis. The values were calculated in relation to the total number of raw reads. Exact duplicate reads were omitted from further analysis. The proportions of reads derived from SSU rRNA, LSU rRNA, and non-rRNA were calculated in relation to the total number of preprocessed reads.
(DOCX)

S2 Table Statistics of functional metatranscriptome libraries. The proportions of exact duplicate and preprocessed reads were calculated in relation to the total number of raw reads. Exact duplicate reads were omitted from further analysis. The proportions of reads derived from rRNA, small RNA, and putative mRNA were calculated in relation to the total number of preprocessed reads.
(DOCX)

## References

[1] Cho J, Tiedje JM. Biogeography and degree of endemicity of fluorescent *Pseudomonas* strains in soil biogeography. Appl Environ Microbiol 2000;66: 5448–5456. 11097926 10.1128/aem.66.12.5448-5456.2000 PMC92480

[2] Crump BC, Hopkinson CS, Sogin ML, Hobbie JE. Microbial biogeography along an estuarine salinity gradient: combined influences of bacterial growth and residence time. Appl Environ Microbiol 2004;70: 1494–1505. 15006771 10.1128/AEM.70.3.1494-1505.2004 PMC365029

[3] García-Martínez J, Rodríguez-Valera F. Microdiversity of uncultured marine prokaryotes: the SAR11 cluster and the marine Archaea of Group I. Mol Ecol 2000;9: 935–948. 10886656 10.1046/j.1365-294x.2000.00953.x

[4] Chaffron S, Rehrauer H, Pernthaler J, von Mering C. A global network of coexisting microbes from environmental and whole-genome sequence data. Genome Res 2010;20: 947–959. 10.1101/gr.104521.109 20458099 PMC2892096

[5] Faust K, Sathirapongsasuti JF, Izard J, Segata N, Gevers D, Raes J, et al Microbial co-occurrence relationships in the human microbiome. PLoS Comput Biol 2012;8: e1002606 10.1371/journal.pcbi.1002606 22807668 PMC3395616

[6] Hubbell SP. Neutral theory and the evolution of ecological equivalence. Ecology 2006;87: 1387–1398. 16869413 10.1890/0012-9658(2006)87[1387:ntateo]2.0.co;2

[7] Vallès Y, Artacho A, Pascual-García A, Ferrús ML, Gosalbes MJ, Abellán JJ, et al Microbial succession in the gut: directional trends of taxonomic and functional change in a birth cohort of Spanish infants. PLoS Genet 2014;10: e1004406 10.1371/journal.pgen.1004406 24901968 PMC4046925

[8] Turnbaugh PJ, Hamady M, Yatsunenko T, Cantarel BL, Duncan A, Ley RE, et al A core gut microbiome in obese and lean twins. Nature 2009;457: 480–484. 10.1038/nature07540 19043404 PMC2677729

[9] Phelan VV, Liu WT, Pogliano K, Dorrestein PC. Microbial metabolic exchange—the chemotype-to-phenotype link. Nat Chem Biol 2012;8: 26–35. 10.1038/nchembio.739 22173357 PMC3869239

[10] Riesenfeld CS, Schloss PD, Handelsman J. Metagenomics: genomic analysis of microbial communities. Annu Rev Genet 2004;38: 525–552. 15568985 10.1146/annurev.genet.38.072902.091216

[11] Moran MA. Metatranscriptomics: Eavesdropping on complex microbial communities. Microbe 2009;7: 329–335.

[12] Carvalhais LC, Dennis PG, Tyson GW, Schenk PM. Application of metatranscriptomics to soil environments. J Microbial Meth 2012;91: 246–251. 10.1016/j.mimet.2012.08.011 22963791

[13] Moran MA, Satinsky B, Gifford SM, Luo H, Rivers A, Chan LK, et al Sizing up metatranscriptomics. ISME J 2013;7: 237–243. 10.1038/ismej.2012.94 22931831 PMC3554401

[14] Kim Y, Wegner C, Liesack W. Soil metatranscriptomics In: Nannipieri P, Pietramellara G, Renella G, editors. Omics in soil science. Norfolk: Caister Academic Press; 2014 pp. 63–93.

[15] Mou X, Vila-Costa M, Sun S, Zhao W, Sharma S, Moran MA. Metatranscriptomic signature of exogenous polyamine utilization by coastal bacterioplankton. Environ Microbiol Rep 2011;3: 798–806. 10.1111/j.1758-2229.2011.00289.x 23761372

[16] Vila-Costa M, Rinta-Kanto JM, Sun S, Sharma S, Poretsky R, Moran MA. Transcriptomic analysis of a marine bacterial community enriched with dimethylsulfoniopropionate. ISME J 2010;4: 1410–1420. 10.1038/ismej.2010.62 20463763

[17] McCarren J, Becker J, Repeta D, Shi Y, Young C, Malmstrom RR, et al Microbial community transcriptomes reveal microbes and metabolic pathways associated with dissolved organic matter turnover in the sea. Proc Natl Acad Sci USA 2010;107: 16420–16427. 10.1073/pnas.1010732107 20807744 PMC2944720

[18] De Menezes A, Clipson N, Doyle E. Comparative metatranscriptomics reveals widespread community responses during phenanthrene degradation in soil. Environ Microbiol 2012;14: 2577–2588. 10.1111/j.1462-2920.2012.02781.x 22625871

[19] Helbling DE, Ackermann M, Fenner K, Kohler H-PE, Johnson DR. The activity level of a microbial community function can be predicted from its metatranscriptome. ISME J 2012;6: 902–904. 10.1038/ismej.2011.158 22094344 PMC3309364

[20] Martiny JBH, Bohannan BJM, Brown JH, Colwell RK, Fuhrman JA, Green JL, et al Microbial biogeography: putting microorganisms on the map. Nat Rev Microbiol 2006;4: 102–112. 16415926 10.1038/nrmicro1341

[21] Liesack W, Schnell S, Revsbech NP. Microbiology of flooded rice paddies. FEMS Microbiol Rev 2000;24: 625–645. 11077155 10.1111/j.1574-6976.2000.tb00563.x

[22] Shrestha PM, Kube M, Reinhardt R, Liesack W. Transcriptional activity of paddy soil bacterial communities. Environ Microbiol 2009;11: 960–970. 10.1111/j.1462-2920.2008.01821.x 19170728

[23] Urich T, Schleper C. The “Double-RNA” approach to simultaneously assess the structure and function of a soil microbial community In: de Bruijin FJ, editor. Handbook of molecular microbial ecology I: Metagenomics and complementary approaches. Hoboken: John Wiley & Sons, Inc; 2011 pp. 587–596.

[24] DeAngelis KM, Firestone MK. Phylogenetic clustering of soil microbial communities by 16S rRNA but not 16S rRNA genes. Appl Environ Microbiol 2012;78: 2459–2461. 10.1128/AEM.07547-11 22286992 PMC3302588

[25] Blazewicz SJ, Barnard RL, Daly RA, Firestone MK. Evaluating rRNA as an indicator of microbial activity in environmental communities: limitations and uses. ISME J 2013;7: 2061–2068. 10.1038/ismej.2013.102 23823491 PMC3806256

[26] Shrestha M, Abraham WR, Shrestha PM, Noll M, Conrad R. Activity and composition of methanotrophic bacterial communities in planted rice soil studied by flux measurements, analyses of *pmoA* gene and stable isotope probing of phospholipid fatty acids. Environ Microbiol 2008;10: 400–412. 10.1111/j.1462-2920.2007.01462.x 18177369

[27] Noll M, Matthies D, Frenzel P, Derakshani M, Liesack W. Succession of bacterial community structure and diversity in a paddy soil oxygen gradient. Environ Microbiol 2005;7: 382–395. 15683399 10.1111/j.1462-2920.2005.00700.x

[28] Mettel C, Kim Y, Shrestha PM, Liesack W. Extraction of mRNA from soil. Appl Env Microbiol 2010;76: 5995–6000. 10.1128/AEM.03047-09 20622132 PMC2935057

[29] Schmieder R, Edwards R. Quality control and preprocessing of metagenomic datasets. Bioinformatics 2011;27: 863–864. 10.1093/bioinformatics/btr026 21278185 PMC3051327

[30] Morgulis A, Gertz EM, Schäffer AA, Agarwala R. A fast and symmetric DUST implementation to mask low-complexity DNA sequences. J Comput Biol 2006;13: 1028–1040. 16796549 10.1089/cmb.2006.13.1028

[31] Gomez-Alvarez V, Teal TK, Schmidt TM. Systematic artifacts in metagenomes from complex microbial communities. ISME J 2009;3: 1314–1317. 10.1038/ismej.2009.72 19587772

[32] Quast C, Pruesse E, Yilmaz P, Gerken J, Schweer T, Yarza P, et al The SILVA ribosomal RNA gene database project: improved data processing and web-based tools. Nucleic Acids Res 2013;41: D590–6. 10.1093/nar/gks1219 23193283 PMC3531112

[33] Caporaso JG, Kuczynski J, Stombaugh J, Bittinger K, Bushman FD, Costello EK, et al QIIME allows analysis of high-throughput community sequencing data. Nat Methods 2010;7: 335–336. 10.1038/nmeth.f.303 20383131 PMC3156573

[34] Nawrocki EP, Kolbe DL, Eddy SR. Infernal 1.0: inference of RNA alignments. Bioinformatics 2009;25: 1335–1337. 10.1093/bioinformatics/btp157 19307242 PMC2732312

[35] Huson DH, Mitra S, Ruscheweyh HJ, Weber N, Schuster SC. Integrative analysis of environmental sequences using MEGAN4. Genome Res 2011;21: 1552–1560. 10.1101/gr.120618.111 21690186 PMC3166839

[36] Parks DH, Beiko RG. Identifying biologically relevant differences between metagenomic communities. Bioinformatics 2010;26: 715–721. 10.1093/bioinformatics/btq041 20130030

[37] Urich T, Lanzén A, Qi J, Huson DH, Schleper C, Schuster SC. Simultaneous assessment of soil microbial community structure and function through analysis of the meta-transcriptome. PLoS ONE 2008;3: e2527 10.1371/journal.pone.0002527 18575584 PMC2424134

[38] Ochman H, Lawrence JG, Groisman EA. Lateral gene transfer and the nature of bacterial innovation. Nature 2000;405: 299–304. 10830951 10.1038/35012500

[39] Du X, Lipman DJ, Cherry JL. Why does a protein’s evolutionary rate vary over time? Genome Biol Evol 2013;5: 494–503. 10.1093/gbe/evt024 23436005 PMC3622301

[40] Frias-Lopez J, Shi Y, Tyson GW, Coleman ML, Schuster SC, Chisholm SW, et al Microbial community gene expression in ocean surface waters. Proc Natl Acad Sci USA 2008;105: 3805–3810. 10.1073/pnas.0708897105 18316740 PMC2268829

[41] Burow LC, Woebken D, Marshall IPG, Lindquist EA, Bebout BM, Prufert-Bebout L, et al Anoxic carbon flux in photosynthetic microbial mats as revealed by metatranscriptomics. ISME J 2013;7: 817–829. 10.1038/ismej.2012.150 23190731 PMC3603402

[42] Chin KJ, Hahn D, Hengstmann U, Liesack W, Janssen PH. Characterization and identification of numerically abundant culturable bacteria from the anoxic bulk soil of rice paddy microcosms. Appl Environ Microbiol 1999;65: 5042–5049. 10543821 10.1128/aem.65.11.5042-5049.1999 PMC91679

[43] Gregory TR, DeSalle R. Comparative genomics in prokaryotes In: Gregory TR, editor. The evolution of the genome. San Diego: Elsevier; 2005 pp. 585–675.

[44] Makarova KS, Aravind L, Galperin MY, Grishin NV, Tatusov RL, Wolf YI, et al Comparative genomics of the Archaea (Euryarchaeota): evolution of conserved protein families, the stable core, and the variable shell. Genome Res 1999;9: 608–628. 10413400

[45] Damon C, Lehembre F, Oger-Desfeux C, Luis P, Ranger J, Fraissinet-Tachet L, et al Metatranscriptomics reveals the diversity of genes expressed by eukaryotes in forest soils. PLoS ONE 2012;7: e28967 10.1371/journal.pone.0028967 22238585 PMC3253082

[46] Wardle DA, Bardgett RD, Klironomos JN, Setälä H, van der Putten WH, Wall DH. Ecological linkages between aboveground and belowground biota. Science 2004;304: 1629–1633. 15192218 10.1126/science.1094875

[47] Fierer N, Jackson RB. The diversity and biogeography of soil bacterial communities. Proc Natl Acad Sci USA 2006;103: 626–631. 16407148 10.1073/pnas.0507535103 PMC1334650

[48] Wang Q, Li H, Post AF. Nitrate assimilation genes of the marine diazotrophic, filamentous cyanobacterium *Trichodesmium* sp. strain WH9601. J Bacteriol 2000;182: 1764–1767. 10692386 10.1128/jb.182.6.1764-1767.2000 PMC94478

[49] Flores E, Frías JE, Rubio LM, Herrero A. Photosynthetic nitrate assimilation in cyanobacteria. Photosynth Res 2005;83: 117–133. 16143847 10.1007/s11120-004-5830-9

[50] Frenzel P, Rothfuss F, Conrad R. Oxygen profiles and methane turnover in a flooded rice microcosm. Biol Fertil Soils 1992;14: 84–89.

[51] Dawid W. Biology and global distribution of myxobacteria in soils. FEMS Microbiol Rev 2000;24: 403–427. 10978544 10.1111/j.1574-6976.2000.tb00548.x

[52] Pradella S, Hans A, Spröer C, Reichenbach H, Gerth K, Beyer S. Characterisation, genome size and genetic manipulation of the myxobacterium *Sorangium cellulosum* So ce56. Arch Microbiol 2002;178: 484–492. 12420170 10.1007/s00203-002-0479-2

[53] Sims GK, Wander MM. Proteolytic activity under nitrogen or sulfur limitation. Appl Soil Ecol 2002;19: 217–221.

[54] Wawrik B, Kerkhof L, Zylstra GJ, Jerome J, Kukor JJ. Identification of unique type II polyketide synthase genes in soil. Appl Environ Microbiol 2005;71: 2232–2238. 15870305 10.1128/AEM.71.5.2232-2238.2005 PMC1087561

[55] Khalil MAK, Rasmussen RA, Shearer MJ. Effects of production and oxidation processes on methane emissions from rice fields. J Geophys Res 1998;103: 25233.

[56] Shi Y, Tyson GW, Eppley JM, DeLong EF. Integrated metatranscriptomic and metagenomic analyses of stratified microbial assemblages in the open ocean. ISME J 2011;5: 999–1013. 10.1038/ismej.2010.189 21151004 PMC3131857

[57] Gottig N, Garavaglia BS, Garofalo CG, Orellano EG, Ottado J. A filamentous hemagglutinin-like protein of *Xanthomonas axonopodis* pv. *citri*, the phytopathogen responsible for citrus canker, is involved in bacterial virulence. PLoS ONE 2009;4: e4358 10.1371/journal.pone.0004358 19194503 PMC2632755

[58] Serkebaeva YM, Kim Y, Liesack W, Dedysh SN. Pyrosequencing-based assessment of the bacteria diversity in surface and subsurface peat layers of a northern wetland, with focus on poorly studied phyla and candidate divisions. PLoS ONE 2013;8: e63994 10.1371/journal.pone.0063994 23700443 PMC3660313

[59] Mills CT, Slater GF, Dias RF, Carr SA, Reddy CM, Schmidt R, et al The relative contribution of methanotrophs to microbial communities and carbon cycling in soil overlying a coal-bed methane seep. FEMS Microbiol Ecol 2013;84: 474–494. 10.1111/1574-6941.12079 23346979

[60] Han B, Chen Y, Abell G, Jiang H, Bodrossy L, Zhao J, et al Diversity and activity of methanotrophs in alkaline soil from a Chinese coal mine. FEMS Microbiol Ecol 2009;70: 40–51. 10.1111/j.1574-6941.2009.00707.x 19515201

[61] Hutchens E, Radajewski S, Dumont MG, McDonald IR, Murrell JC. Analysis of methanotrophic bacteria in Movile Cave by stable isotope probing. Environ Microbiol 2003;6: 111–120. 10.1046/j.1462-2920.2003.00543.x 14756876

[62] Conrad R. Contribution of hydrogen to methane production and control of hydrogen concentrations in methanogenic soils and sediments. FEMS Microbiol Ecol 1999;28: 193–202.

[63] Zhang J, Siika-Aho M, Tenkanen M, Viikari L. The role of acetyl xylan esterase in the solubilization of xylan and enzymatic hydrolysis of wheat straw and giant reed. Biotechnol Biofuels 2011;4: 60 10.1186/1754-6834-4-60 22185437 PMC3259036

[64] Tangney M, Mitchell WJ. Characterisation of a glucose phosphotransferase system in *Clostridium acetobutylicum* ATCC 824. Appl Microbiol Biotechnol 2007;74: 398–405. 17096120 10.1007/s00253-006-0679-9

[65] Yu Y, Tangney M, Aass HC, Mitchell WJ. Analysis of the mechanism and regulation of lactose transport and metabolism in *Clostridium acetobutylicum* ATCC 824. Appl Environ Microbiol 2007;73: 1842–1850. 17209069 10.1128/AEM.02082-06 PMC1828815

[66] Sára M, Sleytr UB. S-layer proteins. J Bacteriol 2000;182: 859–868. 10648507 10.1128/jb.182.4.859-868.2000 PMC94357

[67] Hori T, Noll M, Igarashi Y, Friedrich MW, Conrad R. Identification of acetate-assimilating microorganisms under methanogenic conditions in anoxic rice field soil by comparative stable isotope probing of RNA. Appl Environ Microbiol 2007;73: 101–109. 17071795 10.1128/AEM.01676-06 PMC1797110

[68] Hori T, Müller A, Igarashi Y, Conrad R, Friedrich MW. Identification of iron-reducing microorganisms in anoxic rice paddy soil by ^13^C-acetate probing. ISME J 2010;4: 267–278. 10.1038/ismej.2009.100 19776769

[69] Reguera G, McCarthy KD, Mehta T, Nicoll JS, Tuominen MT, Lovley DR. Extracellular electron transfer via microbial nanowires. Nature 2005;435: 1098–1101. 15973408 10.1038/nature03661

[70] Tremblay PL, Aklujkar M, Leang C, Nevin KP, Lovley D. A genetic system for *Geobacter metallireducens*: role of the flagellin and pilin in the reduction of Fe(III) oxide. Environ Microbiol Rep 2012;4: 82–88. 10.1111/j.1758-2229.2011.00305.x 23757233

[71] Chidthaisong A, Rosenstock B, Conrad R. Measurement of monosaccharides and conversion of glucose to acetate in anoxic rice field soil. Appl Environ Microbiol 1999;65: 2350–2355. 10347012 10.1128/aem.65.6.2350-2355.1999 PMC91347

[72] Stewart FJ, Sharma AK, Bryant JA, Eppley JM, DeLong EF. Community transcriptomics reveals universal patterns of protein sequence conservation in natural microbial communities. Genome Biol 2011;12: R26 10.1186/gb-2011-12-3-r26 21426537 PMC3129676

[73] Cordero OX, Wildschutte H, Kirkup B, Proehl S, Ngo L, Hussain F, et al Ecological populations of bacteria act as socially cohesive units of antibiotic production and resistance. Science 2012;337: 1228–1231. 10.1126/science.1219385 22955834

[74] Burke C, Steinberg P, Rusch D, Kjelleberg S, Thomas T. Bacterial community assembly based on functional genes rather than species. Proc Natl Acad Sci USA 2011;108: 14288–14293. 10.1073/pnas.1101591108 21825123 PMC3161577

[75] Finucane MM, Sharpton TJ, Laurent TJ, Pollard KS. A taxonomic signature of obesity in the microbiome? Getting to the guts of the matter. PLoS ONE 2014;9: e84689 10.1371/journal.pone.0084689 24416266 PMC3885756

