# Supplementary figures and images for: Differential Assemblage of Functional Units in Paddy Soil Microbiomes
Source: PLoS One. 2015 Apr 21;10(4):e0122221. doi: 10.1371/journal.pone.0122221 (PMC4405575; doi:10.1371/journal.pone.0122221)

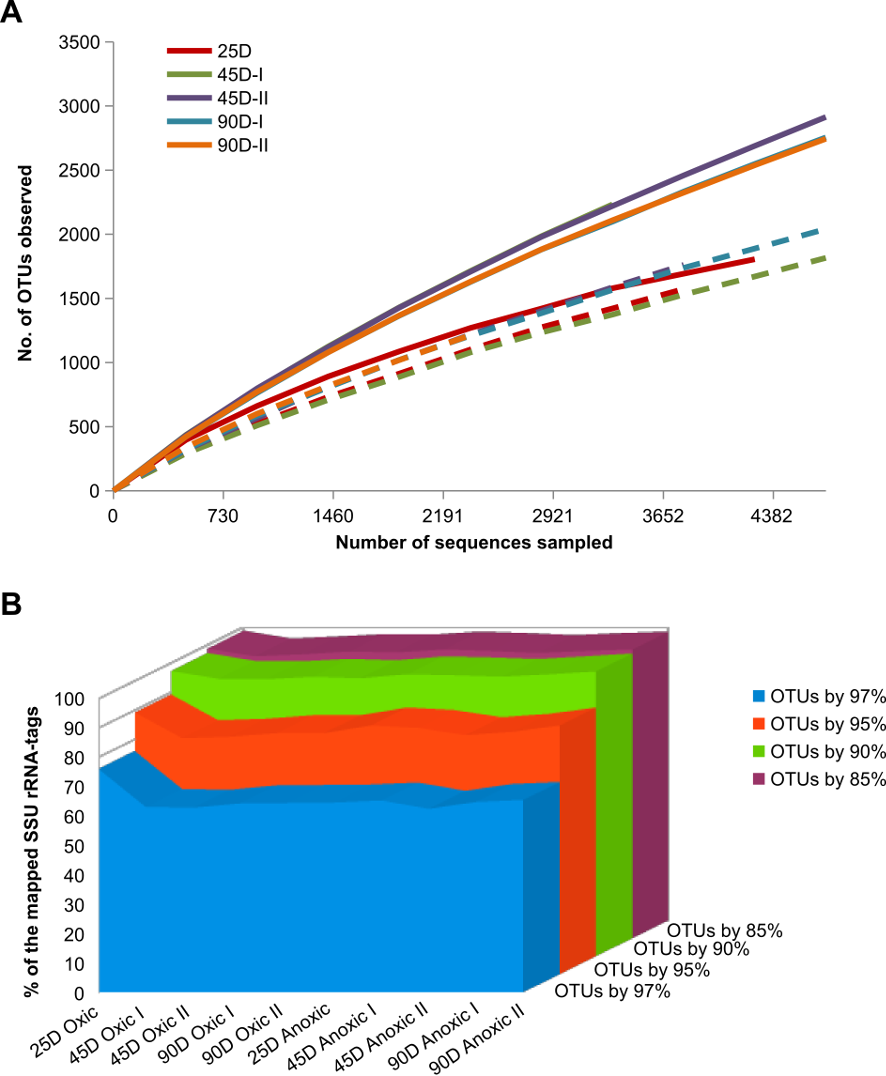

Supplement: S1 Fig — Samples from the oxic and anoxic zones of the same incubation time point are indicated in the same color but differentiated by dashed and solid lines, respectively. Note that some lines are superimposed. (TIF) [file pone.0122221.s001.tif]

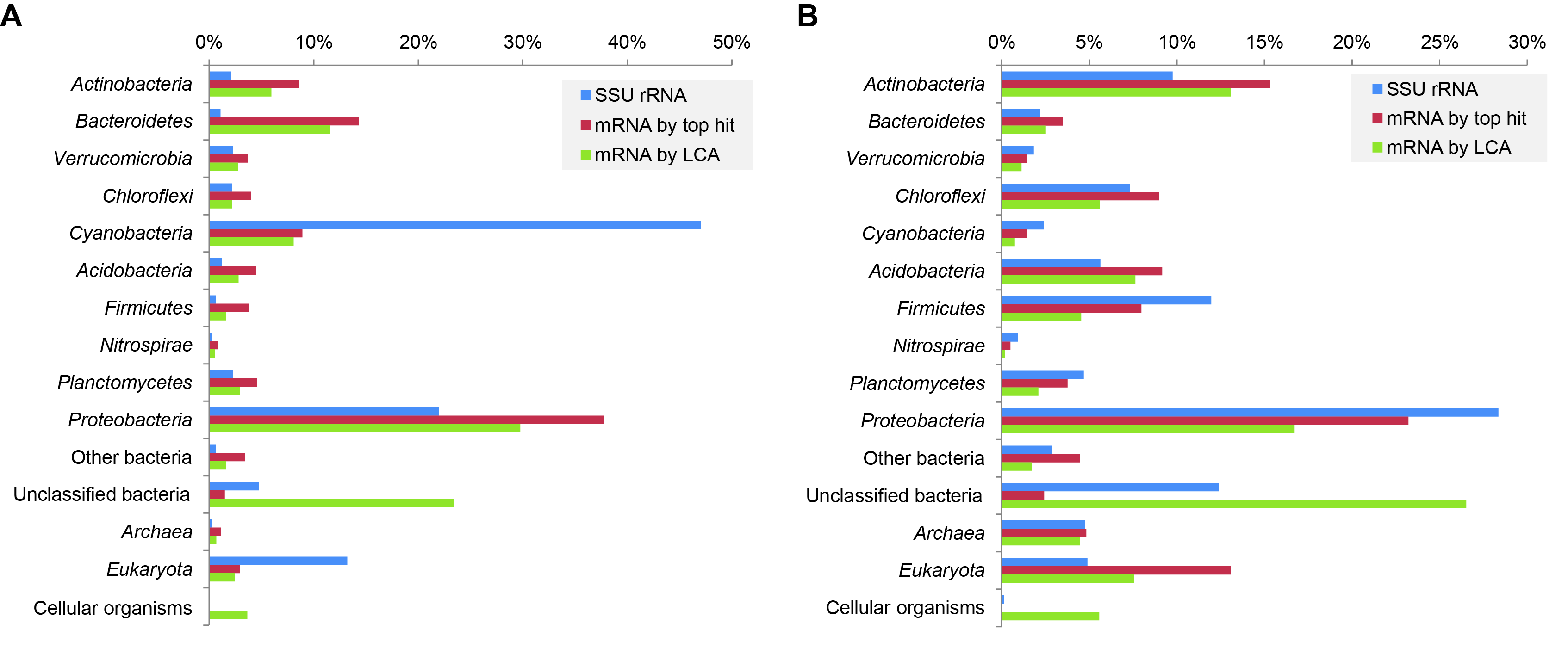

Supplement: S2 Fig — (TIF) [file pone.0122221.s002.tif]

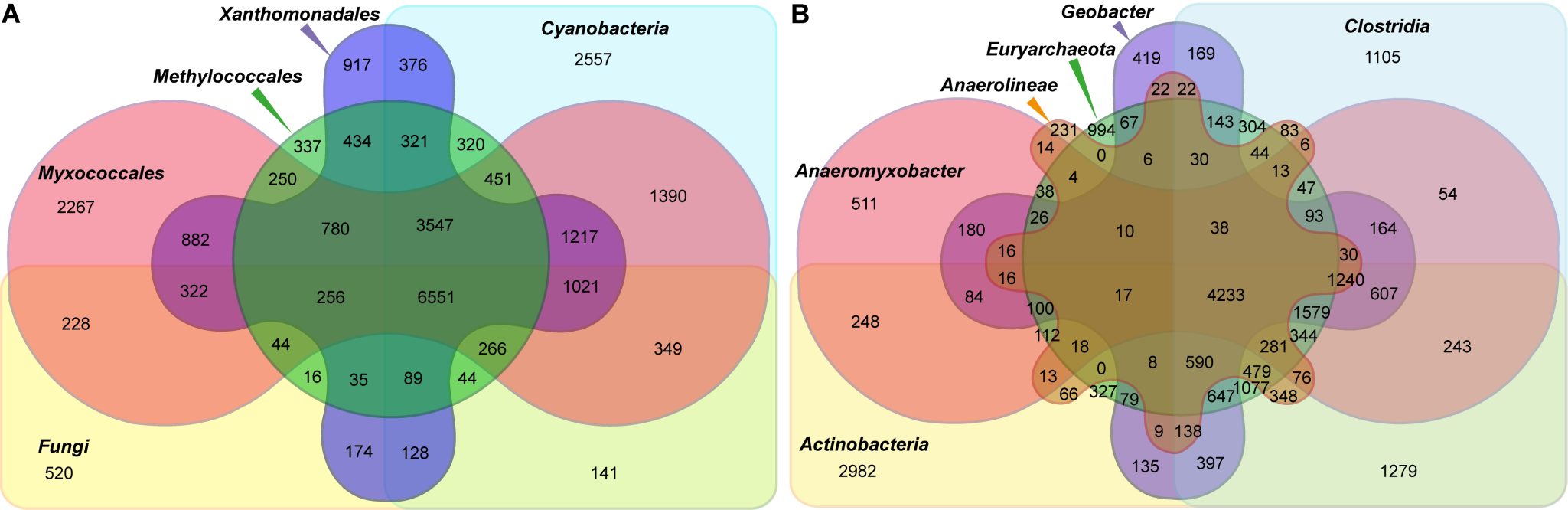

Supplement: S3 Fig — (TIF) [file pone.0122221.s003.tif]

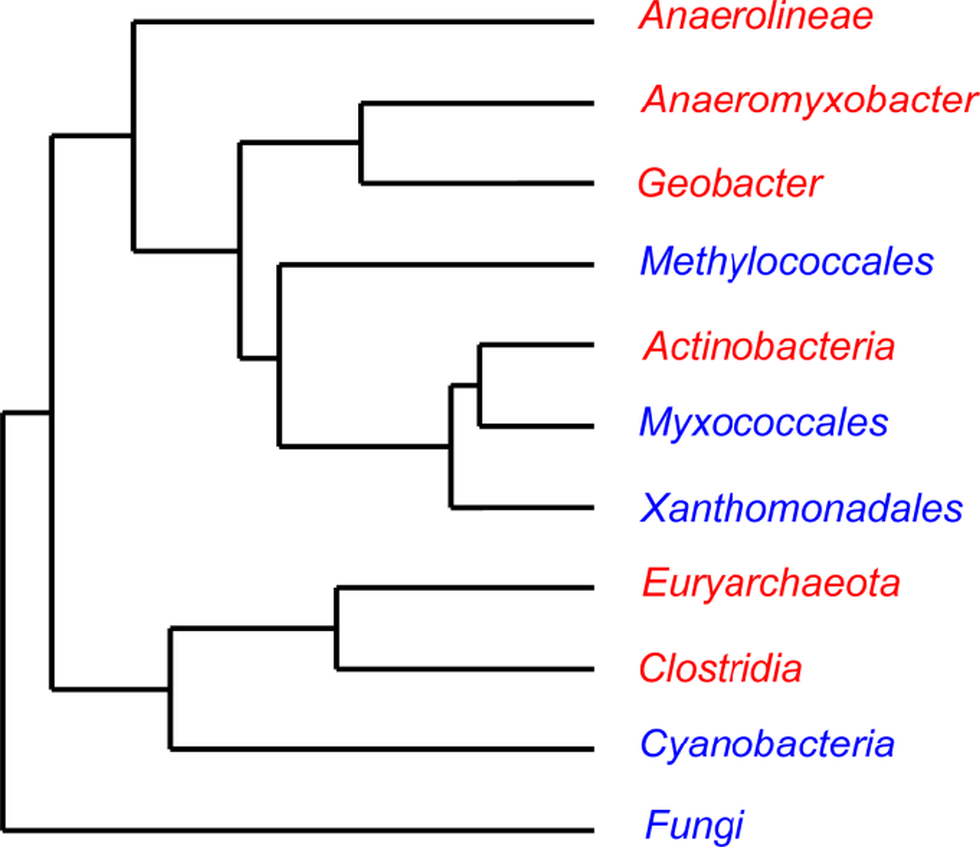

Supplement: S4 Fig — (TIF) [file pone.0122221.s004.tif]

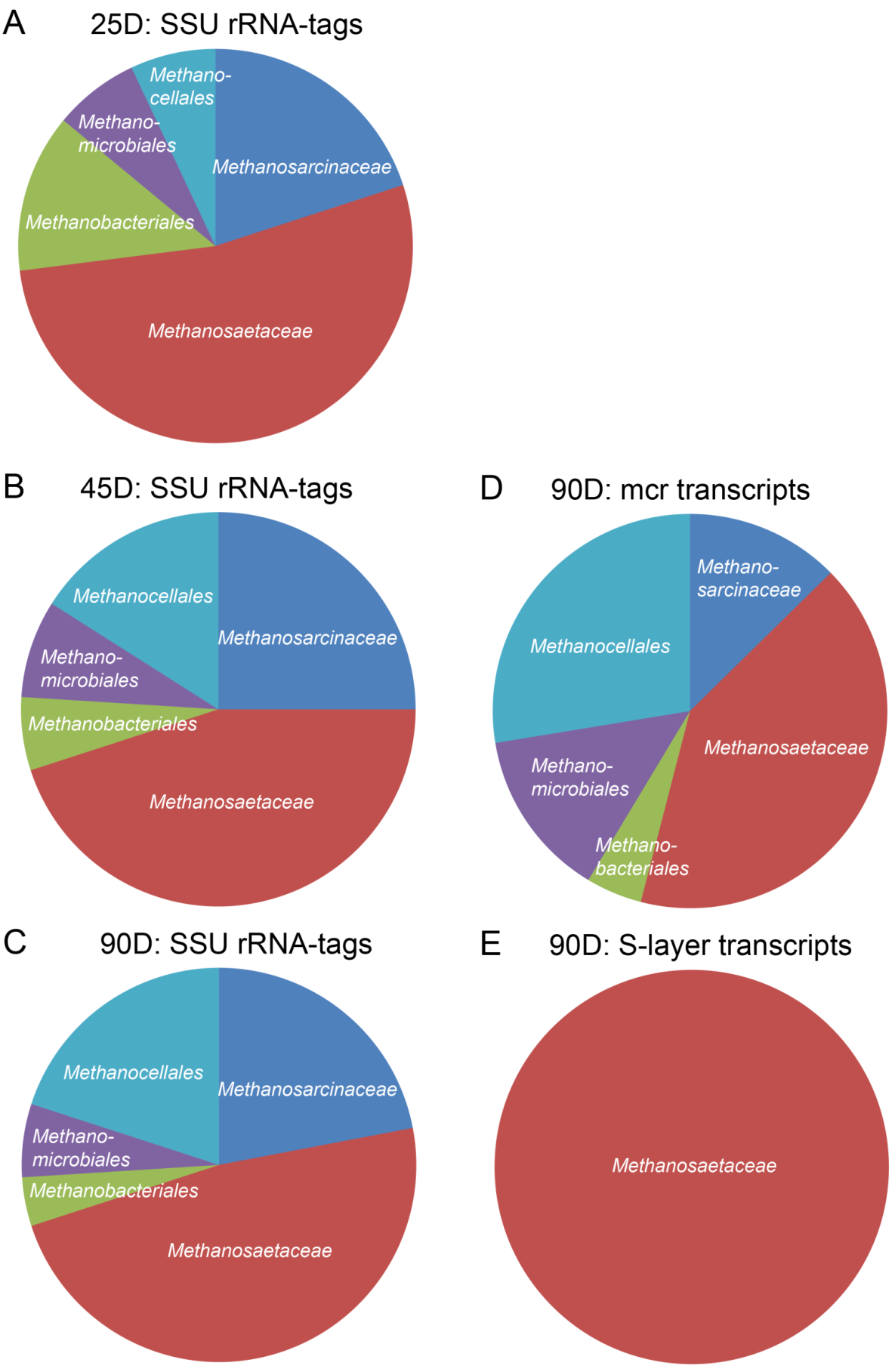

Supplement: S5 Fig — (TIF) [file pone.0122221.s005.tif]
