# Supplementary material for: Differential Assemblage of Functional Units in Paddy Soil Microbiomes
Source: PLoS One. 2015 Apr 21;10(4):e0122221. doi: 10.1371/journal.pone.0122221 (PMC4405575; doi:10.1371/journal.pone.0122221)
Supplement: S1 Table — Total RNA was extracted from two independent replicate microcosms, except for the 25-day incubation period. The proportion of exact duplicates and preprocessed reads is given in parenthesis. The values were calculated in relation to the total number of raw reads. Exact duplicate reads were omitted from further analysis. The proportions of reads derived from SSU rRNA, LSU rRNA, and non-rRNA were calculated in relation to the total number of preprocessed reads. (DOCX) [file pone.0122221.s008.docx]

|  | **Oxic surface layer** | | | | | **Anoxic bulk soil** | | | | |
| --- | --- | --- | --- | --- | --- | --- | --- | --- | --- | --- |
| Number of reads | 25D I | 45D I | 45D II | 90D I | 90D II | 25D I | 45D I | 45D II | 90D I | 90D II |
| Raw reads | 17,249 | 48,987 | 14,006 | 20,441 | 7,528 | 16,567 | 10,430 | 15,902 | 18,169 | 16,707 |
| Exact duplicate reads | 125 (0.72%) | 786 (1.60%) | 150 (1.07%) | 330 (1.61%) | 180 (2.39%) | 79 (0.48%) | 80 (0.77%) | 170 (1.07%) | 81 (0.45%) | 200 (1.18%) |
| Preprocessed reads | 13,906 (80.62%) | 33,211 (67.80%) | 10,365 (74.00%) | 16,532 (80.88%) | 6,209 (82.48%) | 12,201 (73.65%) | 8,405 (80.58%) | 12,492 (78.56%) | 13,892 (76.46%) | 13,222 (79.14%) |
| SSU rRNA | 5,237 (37.66%) | 14,186 (42.71%) | 4,950 (47.76%) | 8,334 (50.41%) | 3,144 (50.64%) | 5,160 (42.29%) | 4,089 (48.65%) | 6,189 (49.54%) | 6,042 (43.49%) | 5,824 (44.05%) |
| LSU rRNA | 8,166 (58.72%) | 18,596 (55.99%) | 5,091 (49.12%) | 7,977 (48.25%) | 2,990 (48.16%) | 6,814 (55.85%) | 4,059 (48.29%) | 5,962 (47.73%) | 7,298 (52.53%) | 6,694 (50.63%) |
| non-rRNA | 503 (3.62%) | 429 (1.29%) | 324 (3.13%) | 221 (1.37%) | 75 (1.21%) | 227 (1.86%) | 257 3.06%) | 341 (2.73%) | 552 (3.97%) | 704 (5.32%) |
| Average length of raw reads (bp) | 403.3 | 381.2 | 391.3 | 406.8 | 355.3 | 389.2 | 313.6 | 338.7 | 397.0 | 365.8 |
| Average length of preprocessed reads (bp) | 440.9 | 437.5 | 444.1 | 469.2 | 424.4 | 428.0 | 423.2 | 430.8 | 435.0 | 451.2 |

**S1 Table. Statistics of ribosomal metatranscriptome libraries.** Total RNA was extracted from two independent replicate microcosms, except for the 25-day incubation period. The proportion of exact duplicates and preprocessed reads is given in parenthesis. The values were calculated in relation to the total number of raw reads. Exact duplicate reads were omitted from further analysis. The proportions of reads derived from SSU rRNA, LSU rRNA, and non-rRNA were calculated in relation to the total number of preprocessed reads.
