# Supplementary material for: Differential Assemblage of Functional Units in Paddy Soil Microbiomes
Source: PLoS One. 2015 Apr 21;10(4):e0122221. doi: 10.1371/journal.pone.0122221 (PMC4405575; doi:10.1371/journal.pone.0122221)
Supplement: S2 Table — The proportions of exact duplicate and preprocessed reads were calculated in relation to the total number of raw reads. Exact duplicate reads were omitted from further analysis. The proportions of reads derived from rRNA, small RNA, and putative mRNA were calculated in relation to the total number of preprocessed reads. (DOCX) [file pone.0122221.s009.docx]

| Number of reads | **Oxic surface layer** | **Anoxic bulk soil** |
| --- | --- | --- |
| Raw reads | 73,974 | 464,906 |
| Exact duplicate reads | 12,020 (16.25%) | 16,179 (3.48%) |
| Preprocessed reads | 53,121 (71.8%) | 342,190 (73.6%) |
| rRNA-tags | 4,196 (7.9%) | 249,456 (72.9%) |
| small RNA-tags | 109 (0.2%) | 489 (0.15%) |
| putative mRNA-tags | 48,816 (91.9%) | 92,245 (27.0%) |
| Average length of raw reads (bp) | 369.5 | 455.5 |
| Average length of preprocessed reads (bp) | 553.3 | 480.3 |

**S2 Table. Statistics of functional metatranscriptome libraries.** The proportions of exact duplicate and preprocessed reads were calculated in relation to the total number of raw reads. Exact duplicate reads were omitted from further analysis. The proportions of reads derived from rRNA, small RNA, and putative mRNA were calculated in relation to the total number of preprocessed reads.
